# Local Dynamics of Collaboration for Maternal, Newborn and Child Health: A Social Network Analysis of Healthcare Providers and Their Managers in Gert Sibande District, South Africa

**DOI:** 10.34172/ijhpm.2021.106

**Published:** 2021-09-08

**Authors:** Fidele Kanyimbu Mukinda, Sara Van Belle, Helen Schneider

**Affiliations:** ^1^School of Public Health, University of the Western Cape, Cape Town, South Africa.; ^2^Institute of Tropical Medicine, Antwerp, Belgium.; ^3^South African Medical Research Council Health Services to Systems Unit, University of the Western Cape, Cape Town, South Africa.

**Keywords:** Collaboration, Accountability, District Health System, Social Network Analysis, Quality Improvement

## Abstract

**Background:** Accountability for maternal, newborn and child health (MNCH) is a collaborative endeavour and documenting collaboration dynamics may be key to understanding variations in the performance of MNCH services. This study explored the dynamics of collaboration among frontline health professionals participating in two MNCH coordination structures in a rural South African district. It examined the role and position of actors, the nature of their relationships, and the overall structure of the collaborative network in two sub-districts.

**Methods:** Cross-sectional survey using a social network analysis (SNA) methodology of 42 district and sub district actors involved in MNCH coordination structures. Different domains of collaboration (eg, communication, professional support, innovation) were surveyed at key interfaces (district-sub-district, across service delivery levels, and within teams).

**Results:** The overall network structure reflected a predominantly hierarchical mode of clustering of organisational relationships around hospitals and their referring primary healthcare (PHC) facilities. Clusters were linked through (and dependent on) a combination of district MNCH programme and line managers, identified as central connectors or boundary spanners. Overall network density remained low suggesting potential for strengthening collaborative relationships. Within cluster collaborative patterns (inter-professional and across levels) varied, highlighting the significance of small units in district functioning.

**Conclusion:** SNA provides a mechanism to uncover the nature of relationships and key actors in collaborative dynamics which could point to system strengths and weaknesses. It offers insights on the level of fragmentation within and across small units, and the need to strengthen cohesion and improve collaborative relationships, and ultimately, the delivery of health services.

## Background

**Key Messages**

** Implications for policy makers**

- Governance and accountability mechanisms for maternal, newborn and child health (MNCH) need to recognise the value of collaborative relationships (and informal interactions) between frontline providers and managers, and across levels of care.
- Effective collaborative relationships involve participation and collective decision-making by senior and middle level managers representing both clinical and non-clinical staff.
- Effective collaboration is driven by a multidisciplinary team of actors, with complementary skills and capabilities including doctors, nurses, emergency medical services, allied health workers, health information and administrative staff.
- Referral processes for MNCH depend on effective collaboration between primary healthcare (PHC) facilities and hospitals.

** Implications for the public**

Effective maternal, newborn and child health (MNCH) requires collaboration and networking between hospitals, primary healthcare (PHC) facilities and the community. In these collaborative networks, community voices may be represented through the hospital board, the community-based organisations or other similar mechanisms. However, in South Africa, these collaborative networks are challenged by structural fragmentation, with in particular, little involvement of the community. Opportunities are needed to build cohesion between disparate groups, by creating ties or strengthening existing weak relationships between providers, and supporting active involvement of the community. When frontline health professionals teams are highly interconnected, they are more likely to deliver high-quality care. Once consolidated, collaborative networks will facilitate knowledge transfer, improve referral systems, continuity of care and patient outcomes.

Health systems are social systems that are determined by people who interact through various forms of collaboration or conflict expressed through the sharing of ideas, interests, values, norms, affinities and power. This can be considered the ‘software’ of the health system, a guiding force underpinning the relationships among health system actors and performance.^1^

 The multi-level collaboration and coordination of care between actors in health systems are frequently invoked as key for achieving the Sustainable Development Goals particularly for maternal, neonatal and child health on reducing mortality by ending preventable deaths.^2^

 Collaboration can be viewed as a key attribute of effective governance, enabling knowledge sharing, service coordination and joint problem-solving.^3^ Successful collaboration is built on the recognition of all actors being part of the solution to problems identified, and requires the following: communication skills, trust-building, capabilities for coaching and mentoring, promotion of collective and inclusive decision-making processes that sustain accountability, and equitable practices.^3,4^

 The essence of collaborative networks resides in bringing ‘*disparate groups together so that they can work effectively and synergistically*.’^5^ Collaborative relationships are enabled by or embedded in formal and informal social networks in the work setting^6,7^ and can be affected by differences in professional power, level of expertise and professional and organizational culture.^8^

 A recent systematic review shows that quality improvement collaboratives among frontline providers and managers improve their knowledge, problem-solving skills and collaborative attitude, teamwork and shared leadership.^9^ By enabling synergies among actors involved,^10^ collaboration facilitates collective learning, sharing of experiences and implementation of changes for improved quality of maternal and child healthcare.^11-13^ Through collaboration, a common purpose can be developed and shared in a safe and open environment where actors can freely express their opinions and where diverse viewpoints are encouraged and fairly protected.^14^

 Collaboration is particularly important for frontline providers and managers who are required to coordinate their activities across a variety of interfaces, which include the following interfaces: (*i*)* a professional interface*: within or across group collaboration between doctors, nurses and other professionals in health; (*ii*)* a levels interface*: collaboration across levels of care in a health system including district hospitals, PHC facilities and community based services; (*iii*) a *patient, family and community *interface: between health professionals and communities.^10,12,14^

 Collaborative relationships can be assessed in different ways, from whether actors simply know other relevant people in the network (a pre-requisite for other forms of collaboration), to varying degrees of communication among actors, to particular domains of collaboration, such as professional support mechanisms and opportunities to innovate (sharing new ideas).^15,16^ By enhancing relational ties, professional support mechanisms allow health workers to cope with personal or work-related challenges, and improve the outcomes of health service delivery.^16,17^

 One of the ways to study collaborative interactions between health system actors is through social network analysis (SNA), which provides an understanding of the behaviour of actors involved in a network, and points to gaps in relationships that are required to strengthen the health system for collective action.^18-20^ For example, SNA has been used to explore health system functioning,^18,21^ to assess the extent of communication between providers involved in a HIV care programme in South Africa^22^ and to describe collaboration among organizations providing HIV treatment, maternal service delivery and workforce strengthening in Uganda.^6^ Mundt et al^23^ used SNA to evaluate the association between team communication and quality of care or costs for patients with cardiovascular disease.

 The South African district health system provides the oversight and coordinating mechanism for community-based services, primary healthcare (PHC) facilities and district hospitals. Collaboration between these levels is through referral processes upwards and downwards.^24^ However, the public health system in South Africa is challenged by fragmentation at the point of implementation, lack of coordination and inadequate referral systems that affect the quality and outcomes of care.^24,25^

 This study aimed to assess the dynamics of collaboration on maternal, newborn and child health (MNCH) within a rural South African district, by exploring and quantifying the structure of the collaborative network as well as the role and position of actors involved in two key district MNCH coordination mechanisms. Different domains of collaborative interactions were considered, namely, the knowledge of other actors in the network, the degree of communication, and relationships of professional support and innovation. Prior qualitative research in the study district had identified collaborative relationships as key to MNCH outcomes and to effective accountability mechanisms.^12,26,27^ However, fragmentation, lack of coordination and inter-professional collaboration within clinical teams (medical, nursing) and with managers from various levels of care were also identified as impeding the quality of service provision.^27^

## Methods

###  Study Setting

 This study was conducted in Gert Sibande district, one of three districts of Mpumalanga province, located in the north-east of South Africa. The district has a population of about 1.1 million, with the vast majority (61%) living in rural areas.^28^ The District health system consists of a network of eight district hospitals, one regional hospital and 76 PHC facilities, distributed among seven sub-districts. Two sub-districts containing three hospitals and associated PHC facilities were purposefully selected for this study.

 A number of evidence-based intervention strategies were implemented in the study district during the 2010-2017 period to address the problem of maternal and child mortality (maternal mortality ratio of 328 per 100 000 births).^29^ A new coordinating and accountability structure, the Monitoring and Response Unit (MRU) was established to complement the existing audit mechanisms, the Perinatal and Child under-five Problem Identification Programmes (PPIP and CHIP, respectively). Collectively these structures brought together managers, clinicians, allied health professionals and information officers from various levels of the healthcare system.^12^

###  Study Design

 We conducted a cross-sectional study of the collaboration networks of frontline providers and managers involved in the three coordination structures – MRU, PPIP and CHIP, considered as a proxy for the MNCH community in Gert Sibande district, Mpumalanga province.

 The following properties are measured in a SNA^19^: (*i*) network structure, which relates firstly to the cohesion or connectedness of the network (density or fragmentation); and secondly, to the shape of the network, including distribution of ties between nodes (actors); and (*ii*) actors’ role and position in the network categorized as central highly connected actors and peripheral actors with loose ties.^30^ Granovetter’s ‘the strength of weak ties’ theory was used to explain the dynamics of collaboration.^31^

 Based on their position and level of influence in the network connectivity, actors can be either bridges (*facilitate information to reach isolated actors*), boundary spanners (*linking two groups of people defined by functional affiliation, physical location, or hierarchical levels)* or ‘brokers’ (*facilitate the transfer of specialized knowledge between groups).*^5,15^

###  Study Population and Sampling 

 The key informants (n = 42) were purposefully sampled among frontline managers and providers involved with maternal, neonatal and child health and attending the key coordination structures, namely the PPIP/CHIP and MRU meetings. The 42 respondents were from the district office (cluster 1, n = 6), sub-district 1 (cluster 2, n = 10 and cluster 3, n = 13) and sub-district 2, (cluster 4, n = 13). Key informants consisted of the following: district programme and other managers (n = 4), members of the district maternal and child health clinical specialist team (n = 2), hospital *chief executive officers (*CEOs, n = 3), nursing managers (n = 3), operational managers from PHC facilities (n = 2) and hospital unit managers (2), professional nurses (n = 12), medical officers (n = 12), information managers (n = 1) and allied health professionals (n = 1).

###  Data Collection and Analysis

 Data were collected using a pre-tested closed-ended questionnaire (Supplementary file 1) completed by the 42 respondents. Data collection and analysis followed the sequence of steps suggested by Blanchet and James^19^ and Cross and Parker.^15^

####  Identifying and Describing a Set of Actors Strategically Important for the Network (Step 1)

 The first step was to identify all key actors involved in the MRU and the PPIP/CHIP meetings following a ‘roster’ approach (to identifying alters).^32^ We collated the attendance registers of the meetings during our fieldwork (over 16 months) and presented the respondents (egos) with an accumulated list of names (alters) from which they could select. These lists consisted of the names of those occupying the positions listed above with the addition of emergency services personnel and community representatives. During the survey, respondents were allowed to add any missing name to the list.

####  Define Meaningful Relationships Between Actors (Step 2) 

 Meaningful network relationships are those that facilitate action or decision making among actors. Based on our interaction with frontline health professionals, we identified and adapted a number of domains as representing and revealing collaboration in a network from Cross and Parker^15^ (Table 1). A relationship was reported if the respondent (ego) stated it; the reporting of the relationship did not rely on both the ego and alter indicating its existence. Knowledge of other actors was regarded as a pre-requisite for, and degree of communication as an indication of, a relationship. The types of collaborative relationships were then further defined as professional support and innovation. The domains of professional support, according to Mikkola et al^16^ and Button,^33^ drew on the general social support typology of informational, instrumental and emotional support.

**Table 1:** Typology of Meaningful Collaborative Interactions

	**Pre-requisite**		**Type of Collaborative Relations**			
Domains	Knowledge of other actors	Degree of communication	Professional support mechanisms^16,32^			Innovation
			*Informational*	*Instrumenta*l	*Emotional*	Sharing new ideas
			Feedback/Advice	Problem-solving	On personal matters	
Questions	*I know this person*	*How often do you communicate with each person regarding MNCH issues?*	*I receive feedback from this person/I feel personally comfortable asking this person for advice on work-related matters*	*Who do you turn to for help in solving a problem in your work?*	*Who do you turn to for support on personal matters?*	*Who are you likely to turn to for discussing a new innovative idea?*

Abbreviation: MNCH, maternal, newborn and child health.

 For the question on frequency of communication, the respondents had to choose the corresponding number as follows (*0 = never, 1 = once a quarter, 2 = monthly, 3 = weekly, 4 = daily) *to state how often they communicate regarding MNCH. For other non-frequency questions, the respondents had to select by placing a cross on the relevant collaborators with whom they shared a link.

 The second part of the questionnaire explored the background characteristics of the respondents (such as gender, age group, their current position and duration in that position) as well as their perception of the importance of the MRU and PPIP/CHIP programmes in strengthening accountability for MNCH.

 An information sheet with consent form was emailed or shared as a hard copy to help respondents familiarize themselves with the content. During fieldwork, the content of the questionnaire and the ethical considerations were explained to participants by the first author. The questionnaire was not anonymised to allow for coding and analysis, but all respondents were assigned a unique code to protect their confidentiality. The list containing the names and coded nodes are only accessible to the first author. The questionnaire was piloted on selected actors from the three settings and corrected following suggestions by respondents to the pilot.

 The survey took place either in the facility boardroom or in the respondent’s own office. The questionnaire was completed individually with no interference from peers or the researcher. Respondents were allowed to ask questions for clarification if something was not clear.

####  Visually Analyze the Structure of the Network and the Position of the Actors (Step 3)

 The analysis examined (*i*) the structure of the system, (*ii*) the actors in the network and (*iii*) the relationships between actors.^19^

 Survey data were captured into and analysed (demographic and background) using Microsoft Excel^®^ 2019. The Excel matrices of network data saved as comma-delimited values (.csv) were imported into Gephi software version 0.9.2 for network visualisation and analysis.^34^ The graphs (sociograms) were generated for the district as a whole and each of the three clusters (corresponding to a hospital and its networks of referring PHC facilities). Network graphs were generated for different forms of collaboration (communication, professional support, innovation) within clusters – across levels of the health service and between professional groups – and in the district as a whole.

 Various algorithms are embedded in Gephi software version 0.9.2^34^ that allows visualisation and analysis of network properties. In this study, we report the following three measures: degree centrality, betweenness centrality and network density (Box 1).^35^

**Box 1. Definition of Network Measures^34^**

** Degree centrality**

The number of immediate contacts (alters^*^) an actor (ego^*^) has in a network. It is measured by counting the number of alters adjacent to the ego. It emphasizes an actor’s activity.^35^ Central connectors will have higher degree centrality, while the peripheral actor will have the lowest degree centrality. In-degree refers to the number of edges which are coming into a node (vertex); Out-degree to the number of edges which are coming out of a node.

** Betweenness centrality**

Looks at how often an actor is nested between two other actors. It measures how many times an actor sits on the shortest path between two other actors. Emphasis is on the actor’s control over information flow.^35^ Boundary spanner and information broker will therefore have high betweenness centrality. Bridges, however, will reduce the distance between nodes (individuals) in a network enhancing the diffusion of information.^36^

** Density**

The extent to which all possible relations are actually present. It represents the completeness or connectedness of a network.^32^

---------------

* Ego = a focal node that represents a respondent; alter=the nodes to whom the respondent (ego) is directly connected.

Actors were represented by a coded node and relations between actors were denoted with an arrowed directed line (edges) for directed relationships. The size of the node depended on the number of connections (degree centrality) or the number of times an actor was sitting on the shortest path between two actors (betweenness). The visualisation allowed us to identify not only influential central actors that are the most connected but also peripheral actors with loose connections.^19^

## Results

###  Characteristics of Study Respondents

 The total network size consisted of 143 nodes distributed as follows: Cluster 1 (n = 23), 18 names provided in the survey and 5 names added by respondents; Cluster 2 (n = 26), all 26 names included with no additions from respondents; Cluster 3 (n = 41), 37 included in the questionnaire, 4 names added by respondents; Cluster 4 (n = 53), 51 names from attendance registers included in the survey and 2 names added by respondents. Of the 143 identified nodes, 42 (29.4%) completed the survey.

Table 2 presents the characteristics of respondents. Overall, 32 (76%) were female, the majority 30 (71%) aged between 41 and 60 years; 10 (23.8%) were doctors and 24 (57.1%) were nurses; and 19 (45.2%) were in a management position. Concerning participation in meetings, 28 (66.7%) had attended the MRU meetings, while 40 (95.2%) had attended PPIP and CHIP meetings; and the majority perceived that these meetings were important in strengthening accountability (Table 3). Although sample sizes are small and possibly non-representative, respondents in Cluster 4 were more satisfied with current accountability mechanisms (and to report participation) than sub-district Clusters 2 and 3.

**Table 2:** Characteristics of Key Informants (n = 42)

	**No. (%)**
Gender	
Female	32 (76.2)
Male	10 (23.8)
Age groups	
20–30	6 (14.3)
31–40	4 (9.5)
41–50	15 (35.7)
51–60	15 (35.7)
Above 60	2 (4.8)
Category	
Doctors	10 (23.8)
Nurses	24 (57.1)
ComServ doctors	4 (9.5)
Dieticians	2 (4.8)
Information officers	2 (4.8)
Position	
District programme managers^a^	6 (14.3)
Hospital ‘Big five’^b^	7 (16.7)
Hospital ward managers	4 (9.5)
PHC managers	2 (4.8)
Other non-managers	23 (54.8)
Position type	
Permanent	35 (83.5)
Non-permanent	7 (16.7)
Duration in position	
Less than 6 months	3 (7.1)
6 months – <1 year	3 (7.1)
1–3 years	7 (16.7)
4–7 years	8 (19.0)
8–10 years	5 (11.9)
Over 10 years	16 (38.1)
Level of care	
District office	6 (14.3)
District hospital	31 (73.8)
Sub-district office	1 (2.4)
PHC	4 (9.5)
Location	
*District Office*	
Cluster 1	6 (14.3)
*Sub-district 1* ^c^	
Cluster 2	10 (23.8)
Cluster 3	13 (31.0)
*Sub-district 2*	
Cluster 4	13 (31.0)

Abbreviation: PHC, primary healthcare. ^a^ Two of them were DCST members based at a regional hospital. ^b^CEO, Medical manager, Nursing manager, Allied health professionals manager. ^c^Sub-district 1 comprises two district hospitals.

**Table 3:** Perception of Accountability Mechanisms

	**Cluster 1 (n = 6) No. (%)**	**Cluster 2 (n = 10) No. (%)**	**Cluster 3 (n = 13) No. (%)**	**Cluster 4 (n = 13) No. (%)**
**MRU**				
Attending MRU meetings (yes)	6 (100.0)	3 (30.0)	9 (69.2)	10 (76.9)
Important for accountability	6 (100.0)	9 (90.0)	12 (92.3)	12 (92.3)
Low importance	-	-	-	-
Neutral	-	-	-	1 (7.7)
Have not heard about MRU	-	1 (10.0)	1 (7.7)	-
**PPIP/CHIP**				
Attending PPIP/CHIP meetings (yes)	5 (83.3)	10 (100.0)	13 (100.0)	12 (92.3)
Important for accountability	5 (83.3)	9 (90.0)	12 (92.3)	13 (100.0)
Low importance	-	1 (10.0)	-	-
Neutral	1 (16.7)	-	1 (7.7)	-
Do not know about	-	-	-	-
**Satisfaction With Current Accountability**				
Satisfied	2 (33.3)	5 (50.0)	10 (76.9)	12 (92.3)
Dissatisfied	2 (33.3)	5 (50.0)	2 (15.4)	1 (7.7)
Neutral	2 (33.3)	-	1 (7.7)	-

Abbreviations: MRU,Monitoring and Response Unit; PPIP, Perinatal Problem Identification Programme; CHIP, Child under-five Problem Identification Programme.

###  Network Structure, Key Actors and Collaboration Across Key Interfaces

 A summary of network metrics is available (see Supplementary file 2 – Table S1). They related to the six domains explored in this study and are described in the sections below. For each domain, only the five actors with the highest metrics are reported.

 The sections which follow report on the overall network structure and key actors involved in MNCH in the district, followed by examination of collaboration across the key interfaces at sub-district level (professional and service delivery levels). The patterns were very similar across all domains and only four of the six domains are reported in the results – namely, knowledge of other MNCH actors, degree of communication, problem solving and sharing of new ideas. The remainder are available as Supplementary file 3.

####  Network Structure 

**Figure 1:**
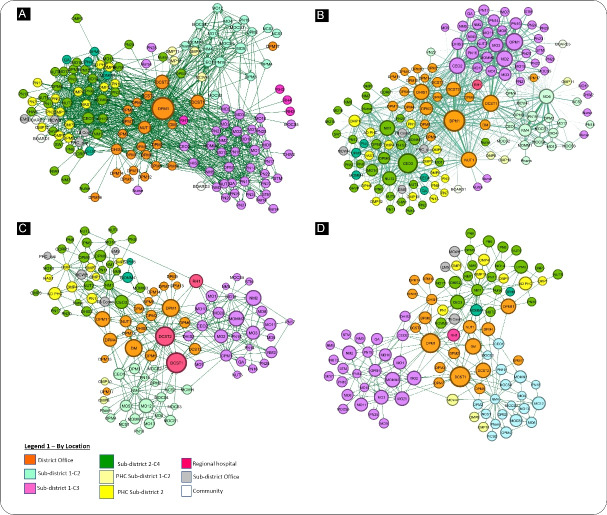
District Level Networks by Location – ‘I Know This Person’ (A), Degree of Communication (B), Problem-Solving (C) and Sharing New Idea (D). Abbreviation: PHC, primary healthcare.

Figure 1 shows the district network as a whole, colour coded by location (district and 3 sub-district clusters) and level (PHC, hospital, community, district), and labelled by actor position. The network structure shows the central cluster (1) of the district office and the three hospital clusters (2-4) around it, connected to other clusters principally through the district office. This clustering reflects the reporting hierarchy in the overall collaborative network. All domains of collaboration, namely, knowledge of other actors, degree of communication, problem-solving or sharing a new idea, followed the same pattern. The degree of communication (how often actors communicate), is shown in the graph by the size of the node and the thickness of the ties (ie, the thicker the tie, the more frequent the communication between actors). Similar patterns were seen in feedback/advice (informational) and emotional support networks (see Supplementary file 3 – Figure S1).

 The overall density of the network in all the domains was very low (less than 10%) implying that less than 10% of all potential connections were actually present at district or sub-district levels, indicating a low level of horizontal and non-hierarchical interactions between and within clusters.

####  Key Actors

 The role and position of actors are key to understanding collaborative relationships. The network structure (Figure 1) showed that the dominant actors in the network – with respect to central connectors and boundary spanners – remained fairly consistent across domains of collaboration. At the district office (cluster 1), the collaboration network revolved around the MNCH coordinator (DPM1), the district clinical specialist team (DCST), the nutrition programme coordinator (NUT1) and the information manager (DHIS1). These were the main drivers of MNCH services with the MNCH coordinator as the most influential and the central connector within cluster 1 and in the district as a whole (across all 4 clusters).

**Figure 2:**
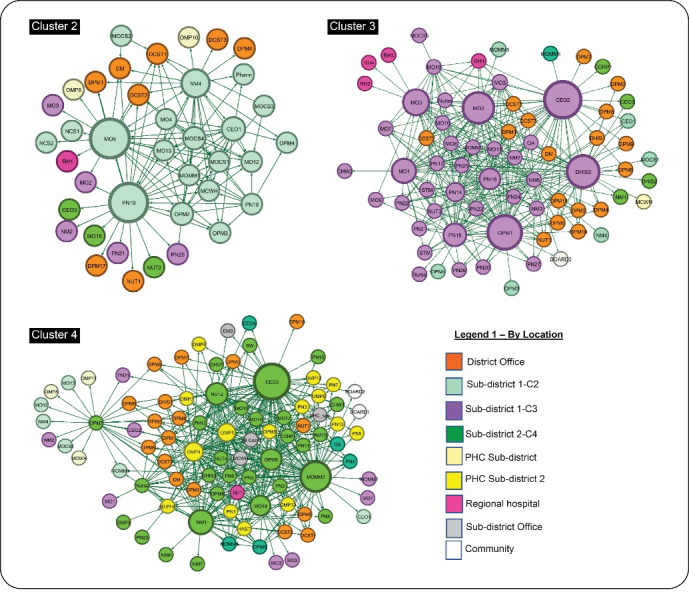
‘I Know This Person’ Network at Sub-district/Cluster Level. Abbreviation: PHC, primary healthcare.

 At sub-district cluster level (Figure 2), there were variations in the position and role of the main actors around whom spun the network, encompassing a mix of influential clinicians, unit nursing managers and members of the hospital management team (referred to as the ‘big five’ – hospital CEO, and medical, nursing, allied health and corporate managers). In cluster 2, the main actors were the medical officer (MO5) from the maternity ward, the professional nurse (PN19) and the nursing manager (NM4). The hospital CEO (CEO1) and the medical manager (MOMM1) also featured in some, although not all, domains (See also online Supplementary file 3 - Figure S2). In cluster 3, five actors were central to the network, namely, the nurse operational manager of maternity ward (OPM1), the CEO (CEO2), the medical officers in maternity and neonatal wards (MO2 and MO3) and the professional nurse in the paediatric ward (PN16). The medical manager (MOMM4) was central in some domains.

**Figure 3:**
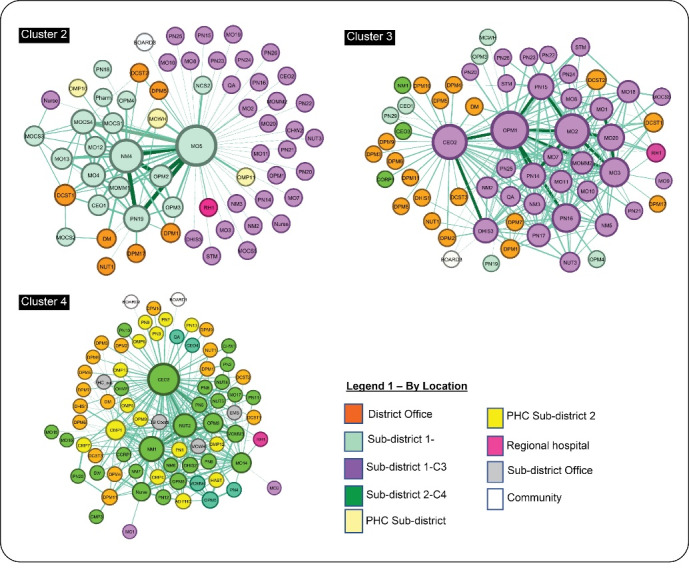
‘Degree of Communication’ Network at Sub-district/Cluster Level. Abbreviation: PHC, primary healthcare.

 The pattern in Cluster 4 (in sub-district 2) was quite different to that of the other two clusters. Here the network revolved most clearly around the designated leadership and in a fairly distributed fashion – the CEO (CEO2), with strong involvement of the nursing service manager (NM1), the allied-health manager (NUT2) and the medical manager (MOMM3). The operational manager paediatric ward (OPM6) also played an influential role (Figure 3).

 The metrics (degree centrality and/or betweenness centrality) for most collaboration domain networks were higher for district actors (district programme managers and DCSTs) as compared to sub-district actors (see Supplementary file 2 – Table S1), illustrating clustering around hierarchies.

 Some variations in the position and role of actors were observed across the domains of collaboration and between clusters. For instance, feedback/advice was provided mostly by the DCSTs and medical officer (cluster 2), the CEO and the maternity ward manager (OPM1) in cluster 3; while in cluster 4, the feedback/advice network consisted of a range of central actors including mid-level nursing managers from both the hospital and PHC facilities.

 The problem-solving network showed that district actors (DCSTs, district manager and the MNCH coordinator) had the highest in-degree values implying that they were the most consulted for problem-solving at district level. At the sub-district level, in addition to consulting actors from the district office, the medical and nursing managers, as well as other medical officers and ward managers, were central in the problem-solving network (clusters 2,3) (see Supplementary file 3 – Figure S3).

 In cluster 4 (sub-district 2) the network showed the central role of the CEO (CEO3) who was also consulted in cluster 3 (sub-district 1) for problem-solving. Also, the involvement of PHC managers who also tend to consult among themselves for solving work-related problems.

####  Collaboration Across Key Interfaces

 This section presents further details regarding collaborative interaction between healthcare levels and professional categories.

####  Collaboration Between Hospitals, PHC Facilities and Community

 There were variations in the patterns of collaboration between the three levels of care (hospital, PHC and community) (Figure 2). Most collaboration happened at the hospital level for all domains. In clusters 2 and 3 there was little or absent engagement of PHC facilities and community representatives. In contrast, in cluster 4, actors from PHC facilities were actively involved in the collaborative network. Communities were represented by the two hospital board chairpersons (BOARD1 and BOARD2) who were known by other actors and were involved in the communication network.

####  Inter-professional Collaboration

 A key feature of collaboration in the district was the clustering around professional categories particularly in the networks related to professional support domains (see online Supplementary file 3 – Figure S2).

 Collaborative relationships for the domains of problem-solving and sharing new ideas (innovation) showed similar patterns between clusters 2 and 3. Doctors and nurses tend to collaborate with each other, the allied health professionals (denoted by ‘other’) collaborating mostly with nurses (see also Supplementary file 3 – Figure S2). The network also depicted the bridging or mediating role of the DCSTs and district (programme) managers.

 Cluster 4 was again the outlier in the pattern of inter-professional collaboration, with greater evidence of a multi-disciplinary team functioning, with the middle-level nursing manager playing a central leadership role (see Supplementary file 1 – Table S2). In the innovation (sharing new ideas), for instance, the network showed involvement of the emergency medical services and PHC managers.

## Discussion

 This paper highlights the value of examining organisational, professional and service delivery relationships and collaboration within a district.

 The network analysis presented in this paper relates to MNCH as a programme that involves ‘many hands,’ that is, an ecosystem of multidisciplinary actors and clusters that contribute to MNCH outcomes seen at system level.^37^ The current organization of healthcare is characterized by vertical reporting lines from PHC and hospitals to the sub-district and district structures. These hierarchical reporting lines are not balanced by mechanisms for horizontal networking and lessons sharing between clusters. In this regard, informal relationships and coordination mechanisms (such as PPIP/CHIP and MRU) present an opportunity to overcome siloes, but require a particular type of local leadership to drive the process.^12,38^

 The overall network revealed strong ties with a few central actors, embedded in a web of absent and weak ties between actors, particularly around the ‘degree of communication’ network. Within the same district, it was expected that there should be a certain level of horizontal collaboration, lesson learning and dissemination across sub-districts, yet the study depicted only limited networking between these clusters. There was thus a dependence on a few central actors who played the role of connectors, bridges or boundary spanners between actors. Because bridges occupy a strategic position in a network, Valente and Fujimoto^36^ argue that any change in the ties from and to the bridging node will reflect on the whole network structure and cohesion.^36^ Dependence on a few critical actors can also create overwhelming workloads for some, making it difficult to respond timeously to needs and demands from below. Referring to central connectors as ‘bottlenecks,’ Cross and Parker^15^ argue that they can hold back the whole network when their capacity to respond is unable to meet the need. Clusters that are highly dependent on central players would be significantly impacted by high turnover of staff and low capacity at central level. Conversely, system resilience could be built by strengthening networks of support and cohesion within and between clusters that do not rely on central mediation.Even weak ties between sub-districts could mitigate the danger of reliance on a few central nodes in a district.

 The significance of DCSTs, district programme managers and other support staff as central actors alongside line managers highlights the interplay of hierarchical and non-hierarchical collaborative relationships. The clinical governance and mentorship roles of programme staff ensured critical links between clusters that otherwise would have remained physically and functionally isolated. They acted as key boundary spanners or bridges with many ties. Some of the boundary spanning actors were formally recognised in their leadership position, while others were not in any formal management position, the so-called ‘unsung heroes’ who played key roles in the network without being officially acknowledged as such.^15^ The roles played by these actors illustrate the vital significance of the informal network in service delivery.

 The actors with the highest betweenness centrality were the district MNCH coordinator, DCSTs, the nutrition and health information managers, the hospital CEOs and some medical officers without any management position. They represented the brokers, sitting on the shortest path between actors, facilitating connections and information flow between levels of care, or translating and adapting higher-level policy initiatives to local needs through clinical leadership and oversight. Long et al^5^ argue that brokers can facilitate transfer of specialised knowledge between disparate groups. By removing the brokers from the network, Cross and Parker^15^ found that the network became more fragmented with many isolated groups. The opposite holds true – increasing the density of ties between disconnected actors will improve efficiency of information diffusion between groups.^5^

 In the study setting, collaboration around MNCH at sub-district level happened mostly within professional categories (doctors, nurses and other professionals including nutrition service and health information managers). There was also variability in the involvement of PHC facilities and community representatives in these clusters, contrary to the findings of a review by David et al^39^ that reported the relevance and participation of PHC professionals and family members in the Brazilian local health system context.

 Overall, the low density or connectedness of the MNCH network suggests a low level of cohesion in the district as a whole and individually in the sub-districts. This was depicted by the high number of absent ties amongst the 42 respondents to the survey (even if a collaborative relationship was recorded when one person in the dyad reported it). Low cohesion between actors within the district can affect the referral systems between actors and across levels of care. It can also explain the disconnect between PHC facilities and district hospitals identified in a previous qualitative phase in the same setting.^27^ Consideration should, therefore, be given to these ‘absent’ and ‘weak ties’ as they represent an opportunity for innovation and strengthening cohesion in a system that is fragmented. Given that the overlap between two individuals’ networks depends on the strength of their tie to one another, focusing only on strong ties, therefore, ignores the potential contribution of ‘weak’ or ‘absent’ ties to system performance.^31^ Granovetter^40^ refers to weak ties as acquaintances as compared to stronger ties of friendship or personal and professional support. Weak ties, when playing a role of local bridges between network segments, can be crucial in generating connectivity between structurally unconnected clusters of a network by facilitating the dissemination of innovative ideas, encouraging inter-cluster communication and collaboration, enhancing productivity and improving health outcomes. Arguing further, Granovetter^40^ suggests that weak ties represent an opportunity for “microintegration” (allowing regular transmission of information) or “macrointegration” (that allows for episodic transmission of information) among disparate or distant clusters that characterise the current healthcare organisation.

 Creating opportunities to strengthen weak ties and reduce absent ties between actors is crucial because when frontline health professionals teams are highly interconnected (higher network density), sharing a common vision with less dependence from the central office, they are more likely to deliver high-quality care.^23^

 Despite similarity of the baseline demographic characteristics across the four Clusters, the data presented in this paper shows that Cluster 4 appeared to provide a model of collaborative relationships for strengthening MNCH and building resilience. Such a model involves the following attributes:

- Firstly, distributed leadership among the ‘big 5’ that creates the space for inclusion, participation and collective decision-making by including senior and middle level managers representing both clinical and non-clinical staff.
- Secondly, effective collaboration driven by a multidisciplinary team of actors, bringing together complementary skills and capabilities including doctors, nurses, emergency medical services, allied health workers, health information and administrative staff.
- Thirdly, PHC facilities are effectively linked to hospitals. Collaboration enables the establishment of effective referral processes and creation of formal and informal networking between hospitals and PHC facilities.
- Fourthly, communities are represented in the various domains of collaboration network. This includes the hospital board chairpersons as representatives of the communities as well as other community-based organisations that provide voice for users and citizens.
- Fifthly, there is less dependence on the central district players. Frontline professionals and managers display a certain independence from the central management office and are empowered with problem-solving capabilities. This requires both stronger cohesion between units and more integration of peripheral actors within and across clusters.
- Finally, innovation is driven by frontline managers. Collaboration aims to empower frontline professionals to bring forward and share new ideas, and new ways of doing things. This would avoid the dependency on the district players for things that require local solutions.

Findings from previous phases of research showed that when encouraged, actors developed innovative informal collaborative relationships and new ways of doing things, such as the establishment of a high risk clinic within the hospital that did not require any additional resources.^26^ These key features of a collaborative network were also described as drivers of the success in MNCH outcomes in the district through strengthened systems of governance.^12^ Prior qualitative observations in the district identified enabling local contexts of accountability and collective responsibility for MNCH care as requiring an open leadership style, multidisciplinary teamwork, involvement of actors across levels of care and community participation.^12,27^ The extended nature of informal accountability relationships developed by the leadership, particularly in Cluster 4, contributed to strengthening co-operation and trust among actors in the sub-district, promoted innovation, and motivated participation in meetings.^12,26^ As pointed out by David et al,^39^ ‘reaffirming the role of PHC in the care network’ is imperative, but also recognising the central role of the managers, particularly district programme managers in mediating collaborative networks.

###  Limitations

 The PIPP/CHIP and the MRU were two examples of collaboration for MNCH that recognised the value of relationships between frontline providers and managers, and across levels of care. It is possible that this network with its strong central connectors, despite its overall low cohesion, functioned better than other service delivery networks (eg, for tuberculosis or non-communicable diseases). This exploratory study provides only limited explanation in the variations between included clusters. This aspect should be considered in future research that should also seek to explore the linkage between SNA analyses and system performance, as well as use SNA in prospectively evaluating quality improvement collaboratives at local level.

 A methodology such as SNA is not able to capture the multiple daily interactions involved in the relationship between providers and patients and community as clients in the collaborative network. These may seem to be weak ties, but their role and contributions no doubt have an impact on MNCH outcomes. The limited representation of actors from PHC facilities can be considered as a methodological limitation. However, only one person had to report on the tie between two people in order for this to be presented in the SNA as an edge. In addition, because the SNA survey was conducted on a meeting day, efforts were made to contact and remind actors regarding the survey. Thus, if the PHC members had been significant players but absent on the day of the survey, then they could have been reported by others or captured in follow-up processes. The absence of PHC players in the study sample in all likelihood represents a weak or absent collaborative network. It is possible that informal collaborative mechanisms existed outside of the PPIP/CHIP or MRU study population, but the prior phases of research suggest that this is unlikely. Finally, a dissemination workshop was planned to give feedback in the district to validate the findings and explore ways to proactively improve collaboration and cohesion in the district. Unfortunately, this workshop was cancelled due to the Covid-19 pandemic.

## Conclusion

 Collaboration is a prime requirement in health systems and maternal and child health, particularly at the district level where frontline health professionals interface with healthcare users. Consolidated collaborative networks are crucial to facilitate knowledge transfer, improve referral systems, continuity of care and, ultimately, patient outcomes. There is a need to build more cohesion among disparate groups within the district health system by integrating PHC, hospitals and communities. Strengthening collaborative networks among multidisciplinary groups of actors from different levels of care will bring isolated groups to work together as a team toward achieving a common goal of improving MNCH outcomes and reducing avoidable deaths. By identifying and utilizing effectively the connectors, spanners and brokers, managers can use the opportunity to close the gaps in knowledge, skills and capabilities among frontline health professionals.

 Governance structures such as the MRU, if well understood and implemented, can facilitate collaborative network and improve cohesion between a multidisciplinary team of actors and across levels of care^12^ particularly by integrating the missing links between PHC, hospital services and communities.^27^ The design of health system reforms should nurture collaborative relationships, information sharing and strengthened teamwork between frontline providers and with clients.^8^

## Acknowledgements

 The authors are grateful for the support and facilitation received from South African National Department of Health, Mpumalanga Department of Health, the managers and staff of Gert Sibande District and Dr. Joey Cupido. We also acknowledge Prof. Asha George for providing critical comments in the conception and analysis of this study.

## Ethical issues

 This paper is part of the PhD study of the first author that was approved by the Biomedical Science Research Ethics Committee of the University of the Western Cape (Reference number: BM17/10/8) and by the Mpumalanga Provincial Health Research Committee (Reference number MP_201801_004).

## Competing interests

 Authors declare that they have no competing interests.

## Authors’ contributions

 FKM designed the study with input from HS and SVB. FKM acquired and analysed the data. FKM, SVB, and HS interpreted the data. FKM drafted the manuscript with input from SVB and HS. All authors made critical revisions of the manuscript and approved the final version.

## Funding

 This work was supported by the funds from the Belgian Development Cooperation, through the Institute of Tropical Medicine Antwerp (Grant Ref: FA4 DGD-ITM 2017-2020). The authors would also like to acknowledge funding from the UWC/SAMRC Health Services to Systems Research Unit and the South African Research Chairs Initiative of the Department of Science and Technology and National Research Foundation of South Africa (grant no. 98918). SVB is paid by senior postdoc fellowship from FWO Belgium (grant no. 1221821N).

## Supplementary files


Supplementary file 1. Social Network Analysis Survey.

Supplementary file 2 contains Tables S1-S2.

Supplementary file 3 contains Figures S1-S3.

## References

[1] Sheikh K, George A, Gilson L (2014). People-centred science: strengthening the practice of health policy and systems research. Health Res Policy Syst.

[2] UN General Assembly. Transforming Our World: the 2030 Agenda for Sustainable Development. United Nations; 2015:1-35.

[3] Torfing J, Peters BG, Pierre J, Sørensen E. Interactive Governance: Advancing the Paradigm. USA: Oxford University Press; 2012.

[4] Melo V. Collaborative Efforts for Sustainable Development: Surveying the Literature on Multi-Stakeholder Initiatives to Realize the Sustainable Development Goals. Task Team on CSO Development Effectiveness and Enabling Environment; 2018. 10.13140/rg.2.2.19706.75209.

[5] Long JC, Cunningham FC, Braithwaite J (2013). Bridges, brokers and boundary spanners in collaborative networks: a systematic review. BMC Health Serv Res.

[6] Ssengooba F, Kawooya V, Namakula J, Fustukian S (2017). Application of social network analysis in the assessment of organization infrastructure for service delivery: a three district case study from post-conflict northern Uganda. Health Policy Plan.

[7] Srinivasan U, Uddin S. A social network framework to explore healthcare collaboration. In: Healthcare Informatics and Analytics: Emerging Issues and Trends. IGI Global; 2014.

[8] Steihaug S, Johannessen AK, Ådnanes M, Paulsen B, Mannion R (2016). Challenges in achieving collaboration in clinical practice: the case of Norwegian health care. Int J Integr Care.

[9] Zamboni K, Baker U, Tyagi M, Schellenberg J, Hill Z, Hanson C (2020). How and under what circumstances do quality improvement collaboratives lead to better outcomes? a systematic review. Implement Sci.

[10] Kilo CM (1999). Improving care through collaboration. Pediatrics.

[11] Lannon CM, Peterson LE (2013). Pediatric collaborative networks for quality improvement and research. Acad Pediatr.

[12] Schneider H, George A, Mukinda F, Tabana H (2020). District governance and improved maternal, neonatal and child health in South Africa: pathways of change. Health Syst Reform.

[13] Waiswa P, Manzi F, Mbaruku G (2017). Effects of the EQUIP quasi-experimental study testing a collaborative quality improvement approach for maternal and newborn health care in Tanzania and Uganda. Implement Sci.

[14] Clemmer TP, Spuhler VJ, Berwick DM, Nolan TW (1998). Cooperation: the foundation of improvement. Ann Intern Med.

[15] Cross R, Parker A. The Hidden Power of Social Networks: Understanding how Work Really Gets Done in Organizations. USA: Harvard Business Review Press; 2004.

[16] Mikkola L, Suutala E, Parviainen H (2018). Social support in the workplace for physicians in specialization training. Med Educ Online.

[17] Hopkinson JB, Hallett CE, Luker KA (2005). Everyday death: how do nurses cope with caring for dying people in hospital?. Int J Nurs Stud.

[18] De Brún A, McAuliffe E (2018). Social network analysis as a methodological approach to explore health systems: a case study exploring support among senior managers/executives in a hospital network. Int J Environ Res Public Health.

[19] Blanchet K, James P (2012). How to do (or not to do) a social network analysis in health systems research. Health Policy Plan.

[20] Braithwaite J (2010). Between-group behaviour in health care: gaps, edges, boundaries, disconnections, weak ties, spaces and holes A systematic review. BMC Health Serv Res.

[21] Chambers D, Wilson P, Thompson C, Harden M (2012). Social network analysis in healthcare settings: a systematic scoping review. PLoS One.

[22] Kawonga M, Blaauw D, Fonn S (2015). Exploring the use of social network analysis to measure communication between disease programme and district managers at sub-national level in South Africa. Soc Sci Med.

[23] Mundt MP, Gilchrist VJ, Fleming MF, Zakletskaia LI, Tuan WJ, Beasley JW (2015). Effects of primary care team social networks on quality of care and costs for patients with cardiovascular disease. Ann Fam Med.

[24] Malakoane B, Heunis JC, Chikobvu P, Kigozi NG, Kruger WH (2020). Public health system challenges in the Free State, South Africa: a situation appraisal to inform health system strengthening. BMC Health Serv Res.

[25] Maphumulo WT, Bhengu BR (2019). Challenges of quality improvement in the healthcare of South Africa post-apartheid: a critical review. Curationis.

[26] Mukinda FK, Van Belle S, George A, Schneider H (2020). The crowded space of local accountability for maternal, newborn and child health: a case study of the South African health system. Health Policy Plan.

[27] Mukinda FK, Van Belle S, Schneider H (2020). Perceptions and experiences of frontline health managers and providers on accountability in a South African health district. Int J Equity Health.

[28] Massyn N, Padarath A, Peer N, Day C. District Health Barometer 2016–2017. Durban: Health Systems Trust; 2017.

[29] Bergh AM, Bac M, Pattinson RC (2019). Changing priorities in maternal and perinatal health in Gert Sibande district, South Africa. S Afr Med J.

[30] Valente TW, Pumpuang P (2007). Identifying opinion leaders to promote behavior change. Health Educ Behav.

[31] Granovetter MS (1973). The strength of weak ties. Am J Sociol.

[32] Scott J. Social Network Analysis. 4th ed. London: SAGE Publications; 2017.

[33] Button LA (2008). Effect of social support and coping strategies on the relationship between health care-related occupational stress and health. J Res Nurs.

[34] GEPHI – Introduction to network analysis and visualization. 2015. http://www.martingrandjean.ch/gephi-introduction. Accessed June 2020.

[35] Prell C. Social Network Analysis: History, Theory and Methodology. London: SAGE Publications; 2012.

[36] Valente TW, Fujimoto K (2010). Bridging: locating critical connectors in a network. Soc Networks.

[37] Dixon-Woods M, Pronovost PJ (2016). Patient safety and the problem of many hands. BMJ Qual Saf.

[38] Mukinda FK, George A, Van Belle S, Schneider H (2021). Practice of death surveillance and response for maternal, newborn and child health: a framework and application to a South African health district. BMJ Open.

[39] David HM, de Araújo Faria MG, Dias JA, da Silva TF, Souza VM, dos Santos Dias R (2018). Social network analysis in primary health care: an integrative review. Acta Paul Enferm.

[40] Granovetter M (1983). The strength of weak ties: a network theory revisited. Sociol Theory.

