# Supplementary material for: Local Dynamics of Collaboration for Maternal, Newborn and Child Health: A Social Network Analysis of Healthcare Providers and Their Managers in Gert Sibande District, South Africa
Source: Int J Health Policy Manag. 2021 Sep 8;11(10):2135–45. doi: 10.34172/ijhpm.2021.106 (PMC9808286; doi:10.34172/ijhpm.2021.106)
Supplement: Supplementary file 1 — Social Network Analysis Survey. [file ijhpm-11-2135-s001.pdf]

Social Network Analysis Survey

Article title: Local Dynamics of Collaboration for Maternal, Newborn and Child Health: A Social Network Analysis of Healthcare Providers and Their Managers in Gert Sibande District, South Africa

Journal name: International Journal of Health Policy and Management (IJHPM)

Authors' information: Fidele Kanyimbu Mukinda[1]*, Sara Van Belle[2], Helen Schneider[1,3]

[1]School of Public Health, University of the Western Cape, Cape Town, South Africa.

[2]Institute of Tropical Medicine, Antwerp, Belgium.

[3]South African Medical Research Council Health Services to Systems Unit, University of the Western Cape, Cape Town, South Africa.

(*Corresponding author: fmukinda@uwc.ac.za)

Supplementary file 1. Social Network Analysis Survey

Introduction

The interviews and field observation we recently had in the district regarding "Accountability for Maternal, Newborn, and Child Health (MNCH)" indicated the need to communicate better and more effectively among actors involved in MNCH processes. We hope that doing some baseline analysis using social network analysis will help us identify what we could do to improve communication, especially in regard to collaboration between all the actors involved in MNCH.

I'm requesting you take some time to provide your input on these questions and send the completed questionnaire to fmukinda@uwc.ac.za (**or hand it in directly**)for analysis (**preferably before 15 July, but sooner would be better**). This should take about 20 minutes of your valuable time to complete.

In this social network analysis, we will try to map the networks for communication, information, problem solving, advice, and support among actors involved with MNCH through Perinatal (PPIP/CHIP) and the Monitoring and Response Unit (MRU) in the District. Social network analysis is useful in visually presenting the relationships between actors (by producing a graph similar to the one at the last page).

Consent

Participation in this study is voluntary, and you may choose not to participate or withdraw your participation in this study at any time. Please note that your answers are confidential. Results that identify you by name will be kept within the research team. In the network maps the names will be replaced with anonymous codes. **If you request**, we will provide you with direct, individualised feedback showing your position in the social network of your district/sub-district.

If you agree to participate in this study you will be asked to answer questions about the people you interact with as well as some background information about you. To map out who communicates with whom, we will request you to give your name in completing this questionnaire.

I'm looking forward to see what we can learn.

Thank you

Dr Fidele Mukinda

Your Consent

I have read, and understood the introduction and the consent parts of this questionnaire. I understand the aim of the research project and what I will be asked to do. I understand that I may stop my participation in this study at any time and that I can refuse to answer any questions.

I also understand that if I take part in the Social Network Analysis I will be required to provide my name which will be replaced with a code.

My Decision: I choose to:	
1	Participate in the study
2	Not to participate in the study

Part One: Social Network Analysis

Your answers to the questions in this first part will allow us to map the network of communication in your Sub-district/District.

Q1. What is your name?

Q2. Where do you work in the District?	*[x]*
☐ District Office	
☐ Sub-district 1	
☐ Sub-district 2	
☐ District Hospital (*Please specify*):	
☐ Clinic (CHC) (*Please specify*):	
☐ Other (*Please specify*)	

Please contact Dr Fidele Mukinda at fmukinda@uwc.ac.za (Cell: xxx-xxxx-xxx) or Prof. Helen Schneider at hschneider@uwc.ac.za for any questions regarding this project.

Q3. In this question, you are presented with a list of people involved in Perinatal or MRU meetings from the District Office, Sub-district 1 and Sub-district 2, and you are requested to please mark with [X] the person you know (Q3)?

*[**Note:** Could you please add names of any person you know that is missing on the list. You can use the next page if you need more space]*

**Q3: I know this person** *[Check all that apply]*								
**District Office**		**Sub-district 1**				**Sub-district 2**	
Name 1		Name 1		Name 1		Name 1	
Name 2		Name 2		Name 2		Name 2	
Name 3		Name 3		Name 3		Name 3	
Name 4		Name 4		Name 4		Name 4	
Other (Specify)		Other (Specify)		Other (Specify)		Other (Specify)	
Other (Specify)		Other (Specify)		Other (Specify)		Other (Specify)	

In the next question (Q4) you are presented with the same list of names of people involved in Perinatal or MRU meetings in GS (**District Office/ Sub-district 1/ Sub-district 2**); you are asked to rate your interactions with each person.

[Note: Please add any other name (Name, title, facility) that is not listed]

	**Q4: How often do you communicate with each person regarding MNCH issues?**<br>0 = Never 1 = Once a quarter 2 = Monthly 3 = Weekly 4 = Daily 99 = I don't know the person	
	Your answers *[Please mark with the corresponding number in each cell]*	
**District Office/ Sub-district 1/ Sub-district 2**		**Q4**
	Name 1	
	Name 2	
	Name 3 ...	
	Other (Name, title, role)	
	Other (Name, title, role)	

Please contact Dr Fidele Mukinda at fmukinda@uwc.ac.za (Cell: xxx-xxxx-xxx) or Prof. Helen Schneider at hschneider@uwc.ac.za for any questions regarding this project.

In the next questions (Q5-Q9) you are presented with the same list of names of people involved in Perinatal or MRU meetings from the **District/Sub-district 1/sub-district 2,** and you are asked to mark with an [X] those related to each question. *[Please select ALL that apply]*

	**List of people**	Q5: I receive feedback from this person?	Q6: I feel personally comfortable asking this person for advice on work-related matters.	Q7: Whom do you turn to for help in solving a problem in your work?	Q8: Who do you turn to for support on personal matters?	Q9: Whom are you likely to turn to discuss a new innovative idea?
		(mark with X)	*(mark with X)*	*(mark with X)*	*(mark with X)*	*(mark with X)*
**District Office/ Sub-district 1/ Sub-district 2**	Name 1					
	Name 2					
	Name 3 …					
	Other (Name, title, role)					
	Other (Name, title, role)					

Please contact Dr Fidele Mukinda at fmukinda@uwc.ac.za (Cell: xxx-xxxx-xxx) or Prof. Helen Schneider at hschneider@uwc.ac.za for any questions regarding this project.

Part Two: Background

1. What is your sex?

☐ Female	1
☐ Male	2

2. Which age group (in years) do you fit into?

☐ Less than 20	1
☐ 20 to 30	2
☐ 31 to 40	3
☐ 41 to 50	4
☐ 51 to 60	5
☐ More than 60	6

3. How long have you worked for the Department of Health? ………………………………
4. What is your current position? (*Mark with X*) *[Choose all that apply]*

☐ CEO		☐ Social worker	
☐ Nursing Manager		☐ Hospital Board chairperson	
☐ Medical Manager		☐ DD MCWYH Coordinator	
☐ Corporate manager		☐ DD Nutrition services	
☐ Sub-district PHC manager		☐ PMTCT Coordinator	
☐ Operational Manager Maternity		☐ DCST (Midwife)	
☐ Operational Manager Paediatrics		☐ DCST (Paeds)	
☐ Operational Manager Clinic/CHC		☐ DCST (O&G)	
☐ Professional Nurse		☐ Quality assurance Manager	
☐ Medical (Paeds)		☐ Pharmacist (District)	
☐ Medical (O&G)		☐ Union Representative	
☐ Medical (Comm Serv)		☐ WBOT	
☐ EMS (Sp)		☐ Other (Please specify)	
☐ Allied Health Manager		☐ Other (Please specify)	
☐ Dietician		☐ Other (Please specify)	
☐ Nutritionist		☐ Other (Please specify)	

Please contact Dr Fidele Mukinda at fmukinda@uwc.ac.za (Cell: xxx-xxxx-xxx) or Prof. Helen Schneider at hschneider@uwc.ac.za for any questions regarding this project.

5. How long have you worked in this position (Q3)?

☐ Less than 6 months	1
☐ 6 months to less than 1 year	2
☐ 1 – 3 years	3
☐ 4 – 7 years	4
☐ 8 – 10 years	5
☐ Over 10 years	6

6. Please indicate whether this position is:

☐ Permanent	1
☐ Acting/delegated	2
☐ Other (Specify):	3

7. Do you [actively] attend this meeting? [*Mark with X*]

	Yes	No
☐ MRU		
☐ Perinatal (CHIP/PPIP)		

8. Do you think the Monitoring and Response Unit (MRU) is important in strengthening accountability for maternal, newborn and child health?

☐ Have not heard of the MRU	1
☐ Not at all important	2
☐ Low importance	3
☐ Neutral	4
☐ Moderately important	5
☐ Very important	6

9. Do you think the perinatal meeting (PPIP & CHIP) is important in strengthening accountability for maternal, newborn and child health?

Please contact Dr Fidele Mukinda at fmukinda@uwc.ac.za (Cell: xxx-xxxx-xxx) or Prof. Helen Schneider at hschneider@uwc.ac.za for any questions regarding this project.

☐ I Don't know	1
☐ Not at all important	2
☐ Low importance	3
☐ Neutral	4
☐ Moderately important	5
☐ Very important	6

10. **INTERESTS**: What are you interested in the most within MNCH? *[Please choose all that apply]*

☐ Perinatal health	1
☐ Neonatal health	2
☐ Child health	3
☐ Nutrition/Malnutrition	4
☐ Maternal health	5
☐ Other (Specify)	6

11. **SATISFACTION**: Overall, how satisfied or dissatisfied are you with your experience with accountability processes regarding MNCH

☐ Very dissatisfied	1
☐ Dissatisfied	2
☐ Neither dissatisfied nor satisfied	3
☐ Satisfied	4
☐ Very satisfied	5

Conclusion & Thank You

Thank you for taking part in this research project and for answering the questions. Your contribution is very much appreciated. Please be assured that your answers will be treated with strict confidence. A feedback session will be held once all data are analyzed.

Please contact Dr Fidele Mukinda at fmukinda@uwc.ac.za (Cell: xxx-xxxx-xxx) or Prof. Helen Schneider at hschneider@uwc.ac.za for any questions regarding this project.

Example of Social Network Analysis Graph[1]

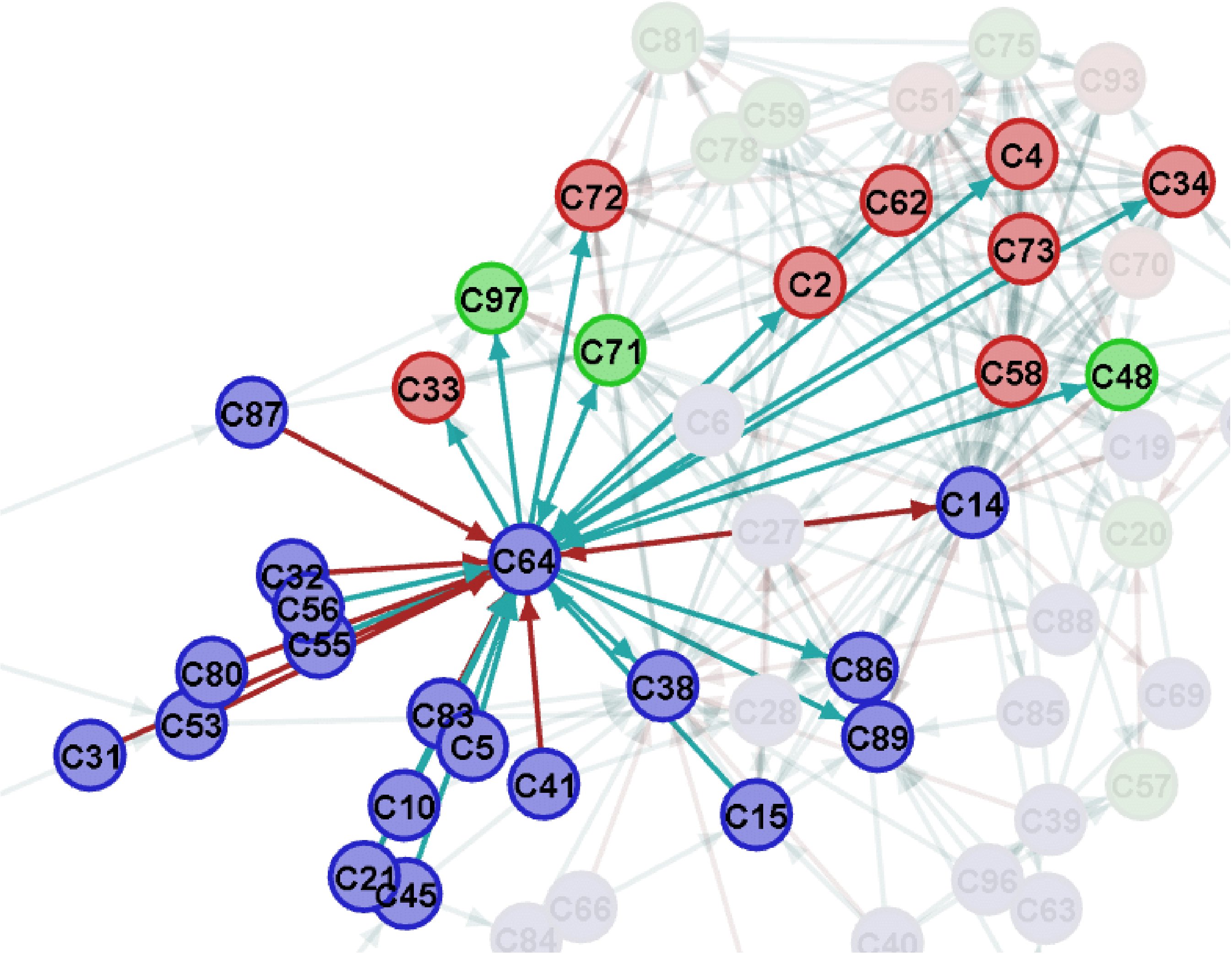

---

[1] Source: Clegg B, Sohal A, Koh C, Dey P, Bennett D. Manufacturing's Wicked Problems (partially) explained through Social Network Analysis2014. doi:10.13140/2.1.3660.6240
