# Supplementary material for: Local Dynamics of Collaboration for Maternal, Newborn and Child Health: A Social Network Analysis of Healthcare Providers and Their Managers in Gert Sibande District, South Africa
Source: Int J Health Policy Manag. 2021 Sep 8;11(10):2135–45. doi: 10.34172/ijhpm.2021.106 (PMC9808286; doi:10.34172/ijhpm.2021.106)
Supplement: Supplementary file 2 — contains Tables S1-S2. [file ijhpm-11-2135-s002.pdf]

Article title: Local Dynamics of Collaboration for Maternal, Newborn and Child Health: A Social Network Analysis of Healthcare Providers and Their Managers in Gert Sibande District, South Africa

Journal name: International Journal of Health Policy and Management (IJHPM)

Authors' information: Fidele Kanyimbu Mukinda[1]*, Sara Van Belle[2], Helen Schneider[1,3]

[1]School of Public Health, University of the Western Cape, Cape Town, South Africa.
[2]Institute of Tropical Medicine, Antwerp, Belgium.
[3]South African Medical Research Council Health Services to Systems Unit, University of the Western Cape, Cape Town, South Africa.

(*Corresponding author: fmukinda@uwc.ac.za)

Supplementary file 2.

Table S1: Network metrics summary

Node metrics	Know other actors			Degree of Communication		
	District	Sub-district 1	Sub-district 2	District	Sub-district 1	Sub-district 2
*Highest betweenness	DPM1 (1540)<br>DCST2 (584)<br>DCST1 (578)<br>NUT1 (419)<br>DHIS1 (217)	MO2 (374)<br>MO5 (324)<br>CEO2 (258)<br>CEO1 (192)<br>PN19 (174)	CEO3 (110)<br>MOMM3 (83)<br>NM1 (53)<br>NUT2 (40)<br>OPM6 (36)	DPM1 (2874)<br>NUT1 (1534)<br>DCST1 (749)<br>DHIS1 (336)<br>DCST2 (333)	MO5 (1210)<br>CEO2 (946)<br>OPM1 (369)<br>NM4 (224)<br>MO3 (170)	CEO3 (1403)<br>NM1 (411)<br>OMP1 (294)<br>NUT2 (243)<br>MO14 (175)
*Highest in-degree	**Degree of Communication****			**Feedback**		
	District	Sub-district 1	Sub-district 2	District	Sub-district 1	Sub-district 2
	DPM1 (228)<br>DCST1 (149)<br>DHIS1 (134)<br>NUT1 (132)<br>DCST2 (113)	OPM1 (156)<br>CEO2 (135)<br>MO2 (135)<br>MO5 (115)<br>MO3 (95)	CEO3 (186)<br>NM1 (117)<br>NUT2 (99)<br>OMP1 (63)<br>MO14 (62)	DCST1 (16)<br>DCST2 (15)<br>DPM1 (14)<br>DPM11 (12)<br>DM (9)	DCST2 (12)<br>DCST1 (12)<br>MOMM2 (10)<br>MO3 (8)<br>MO11 (8)	DPM11 (8)<br>OPM8 (7)<br>AD PHC (6)<br>NM1 (5)<br>OMP4 (5)
	**Advice**			**Problem-solving**		
	District	Sub-district 1	Sub-district 2	District	Sub-district 1	Sub-district 2
	DCST1 (17)<br>DCST2 (16)<br>DPM1 (14)<br>DPM4 (8)<br>DM (8)	DCST1 (12)<br>DCST2 (10)<br>MOMM2 (7)<br>DPM1 (7)<br>NM2 (7)	OPM8 (5)<br>CEO3 (5)<br>AD PHC (5)<br>NUT1 (4)<br>DPM4 (4)	DCST1 (14)<br>DCST2 (13)<br>DM (11)<br>DPM1 (11)<br>DPM11 (9)	DCST1 (10)<br>DCST2 (8)<br>MOMM2 (8)<br>NM2 (8)<br>MO12 (7)	AD PHC (6)<br>OPM8 (5)<br>DPM11 (5)<br>OPM6 (4)<br>CEO3 (4)
	**Emotional support**			**Sharing new idea**		
	District	Sub-district 1	Sub-district 2	District	Sub-district 1	Sub-district 2
	DPM1 (6)<br>DM (5)<br>DCST1 (3)<br>DCST3 (3)<br>DPM5 (2)	NM2 (6)<br>MOMM2 (5)<br>MO11 (5)<br>MO2 (4)<br>DM (4)	OPM8 (3)<br>NM1 (2)<br>AD PHC (2)<br>NUT2 (1)<br>DPM4 (1)	DCST1 (13)<br>DPM1 (11)<br>DM (8)<br>DCST2 (7)<br>DPM11 (7)	DCST1 (10)<br>MOMM2 (6)<br>MO3 (6)<br>DPM1 (6)<br>NM2 (6)	OPM8 (5)<br>DPM11 (5)<br>AD PHC (5)<br>NM1 (3)<br>CEO3 (3)

*Five actors (node code) with the highest metrics (in bracket) are reported. **Weighted degree

Abbreviations: CEO=Chief Executive Officer; DCST=District Clinical Specialist Team; DHIS=District health information system manager; DM=District manager; DPM=District programme manager; MO=Medical officer; MOMM=Medical manager; NM=Nursing services manager; NUT=Nutrition service manager; OMP=Operational manager primary healthcare facility; OPM=Operational manager hospital ward.

Table S2: Degree of communication by professional interface - Actors with highest betweenness centrality*

Cluster 1			Cluster 2			Cluster 3			Cluster 4		
**Doctors**	**Nurses**	**Others**	**Doctors**	**Nurses**	**Others**	**Doctors**	**Nurses**	**Others**	**Doctors**	**Nurses**	**Others**
DCST1 (749)	DPM1 (2874)	NUT1 (1534)	MO5 (1517)	NM4 (220)	-	MO3 (162)	CEO2 (782)	DHIS3 (71)	MO14 (175)	CEO3 (1403)	NUT2 (99)
DCST2 333)	DPM11 (241)	DHIS1 (336)	MOCS4 (18)	PN19 (135)	-	MO2 (150)	OPM1 (514)	NUT3 (0,8)	MOMM3 (15)	NM1 (411)	DHIS2 (20)
-	DPM2 (109)	DPM7 (4)	MO13 (10)	OPM2 (60)	-	MO20 (61)	PN16 (115)	-	MOMM4 (1)	OMP1 (294)	SW (9)
-	DM (94)	DPM3 (1)	MOCS1 (8)	Nurse (6)	-	MO18 (21)	PN15 (81)	-	-	Nurse (98)	NUT4 (9)
-	DCST3 (29)	-	MO12 (8)	CEO1 (1)	-	MOMM2 (8)	PN17 (13)	-	-	OPM6 (70)	NUT5 (9)

*Five actors (node code) with the highest betweenness centrality (in bracket) are reported.

Abbreviations: CEO=Chief Executive Officer; DCST=District Clinical Specialist Team; DHIS=District health information system manager; DM=District manager; DPM=District programme manager; MO=Medical officer; MOMM=Medical manager; NM=Nursing services manager; NUT=Nutrition service manager; OMP=Operational manager primary healthcare facility; OPM=Operational manager hospital ward; SW=Social worker.
