# Supplementary material for: Local Dynamics of Collaboration for Maternal, Newborn and Child Health: A Social Network Analysis of Healthcare Providers and Their Managers in Gert Sibande District, South Africa
Source: Int J Health Policy Manag. 2021 Sep 8;11(10):2135–45. doi: 10.34172/ijhpm.2021.106 (PMC9808286; doi:10.34172/ijhpm.2021.106)
Supplement: Supplementary file 3 — contains Figures S1-S3. [file ijhpm-11-2135-s003.pdf]

Article title: Local Dynamics of Collaboration for Maternal, Newborn and Child Health: A Social Network Analysis of Healthcare Providers and Their Managers in Gert Sibande District, South Africa

Journal name: International Journal of Health Policy and Management (IJHPM)

Authors' information: Fidele Kanyimbu Mukinda[1]*, Sara Van Belle[2], Helen Schneider[1,3]

[1]School of Public Health, University of the Western Cape, Cape Town, South Africa.
[2]Institute of Tropical Medicine, Antwerp, Belgium.
[3]South African Medical Research Council Health Services to Systems Unit, University of the Western Cape, Cape Town, South Africa.

(*Corresponding author: fmukinda@uwc.ac.za)

Supplementary file 3.

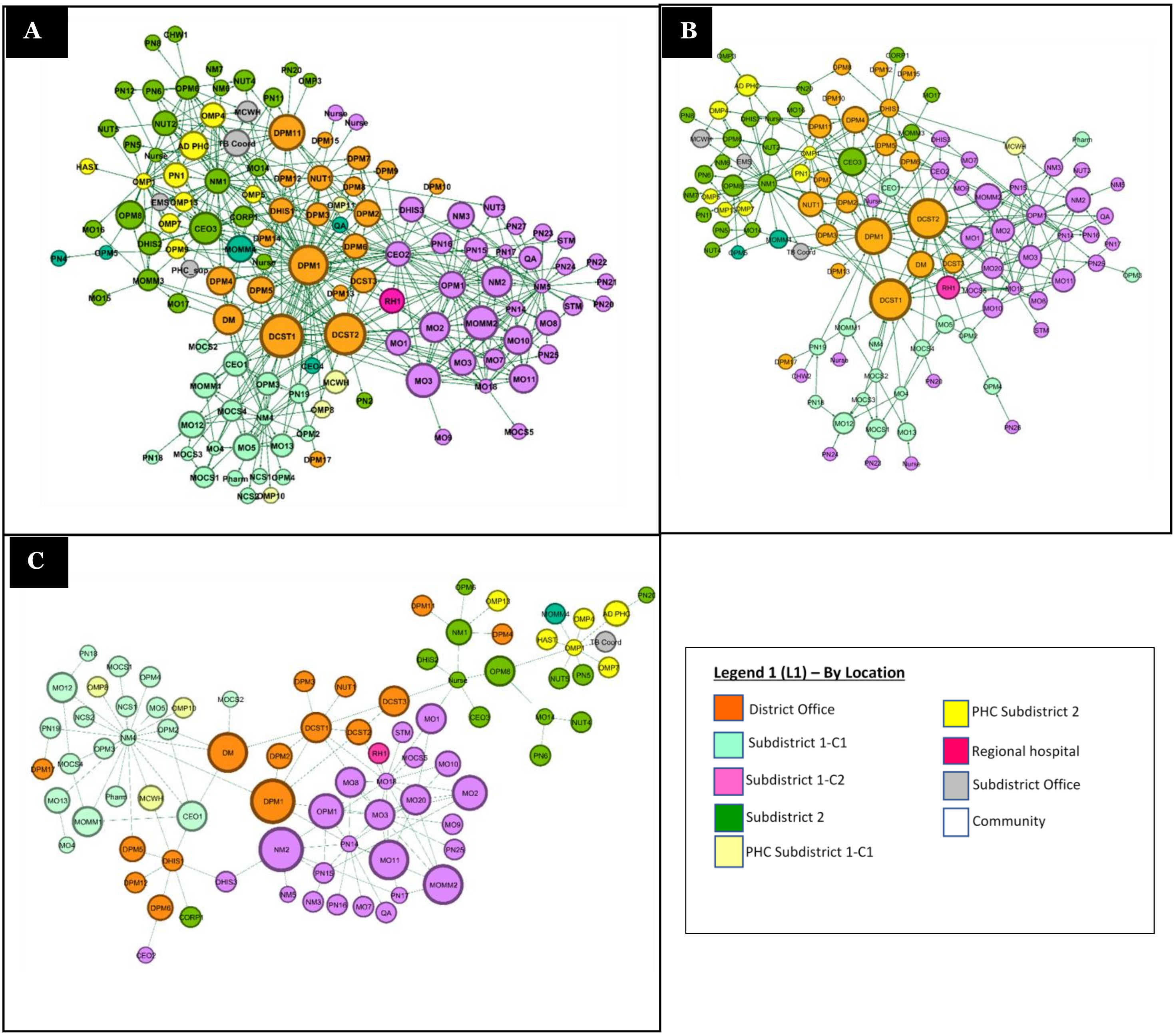

Figure S1: District level – Feedback (A), Advice (B) and Emotional support (C) networks

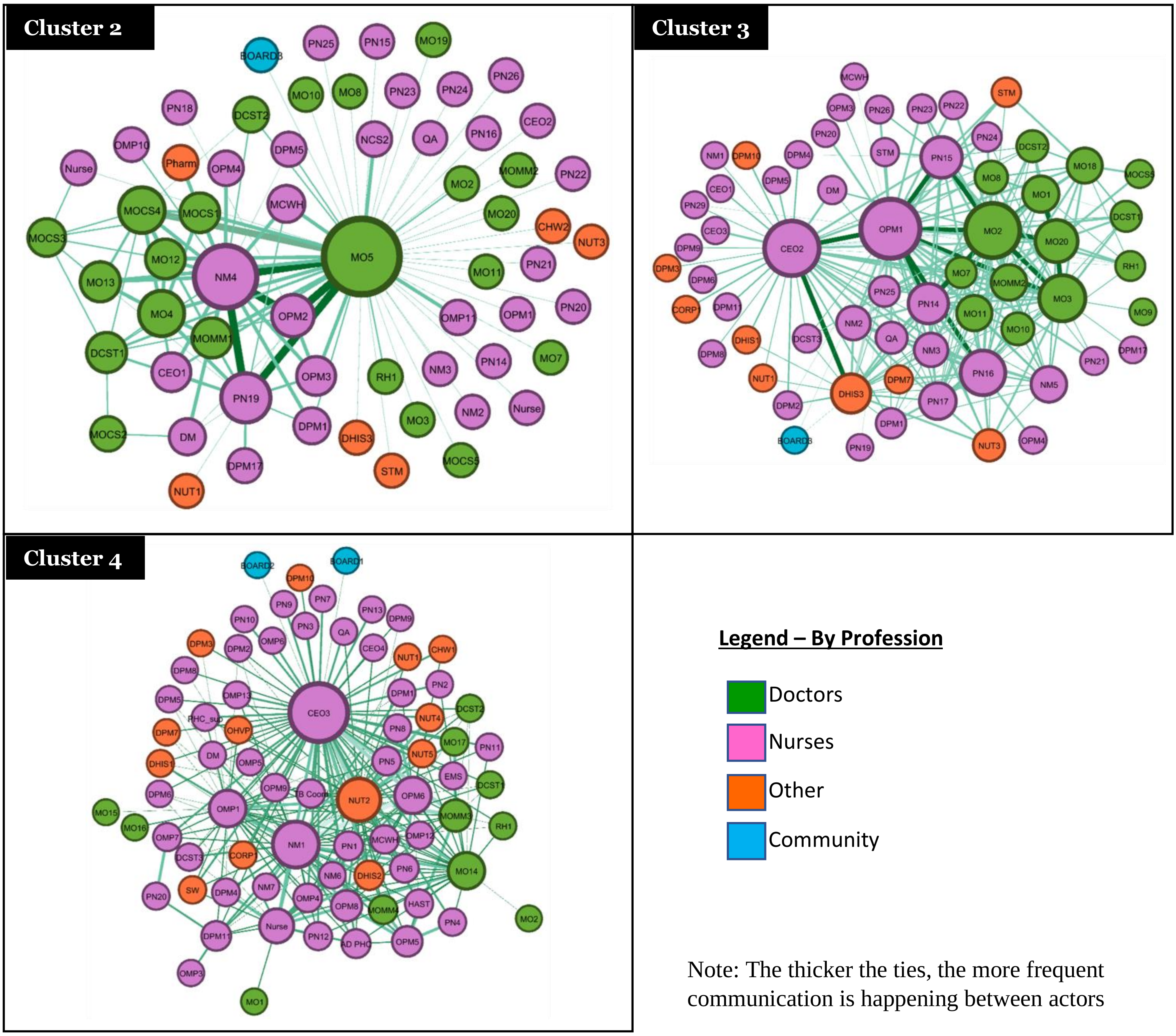

Figure S2: 'Degree of communication' network by professional category at sub-district/cluster level

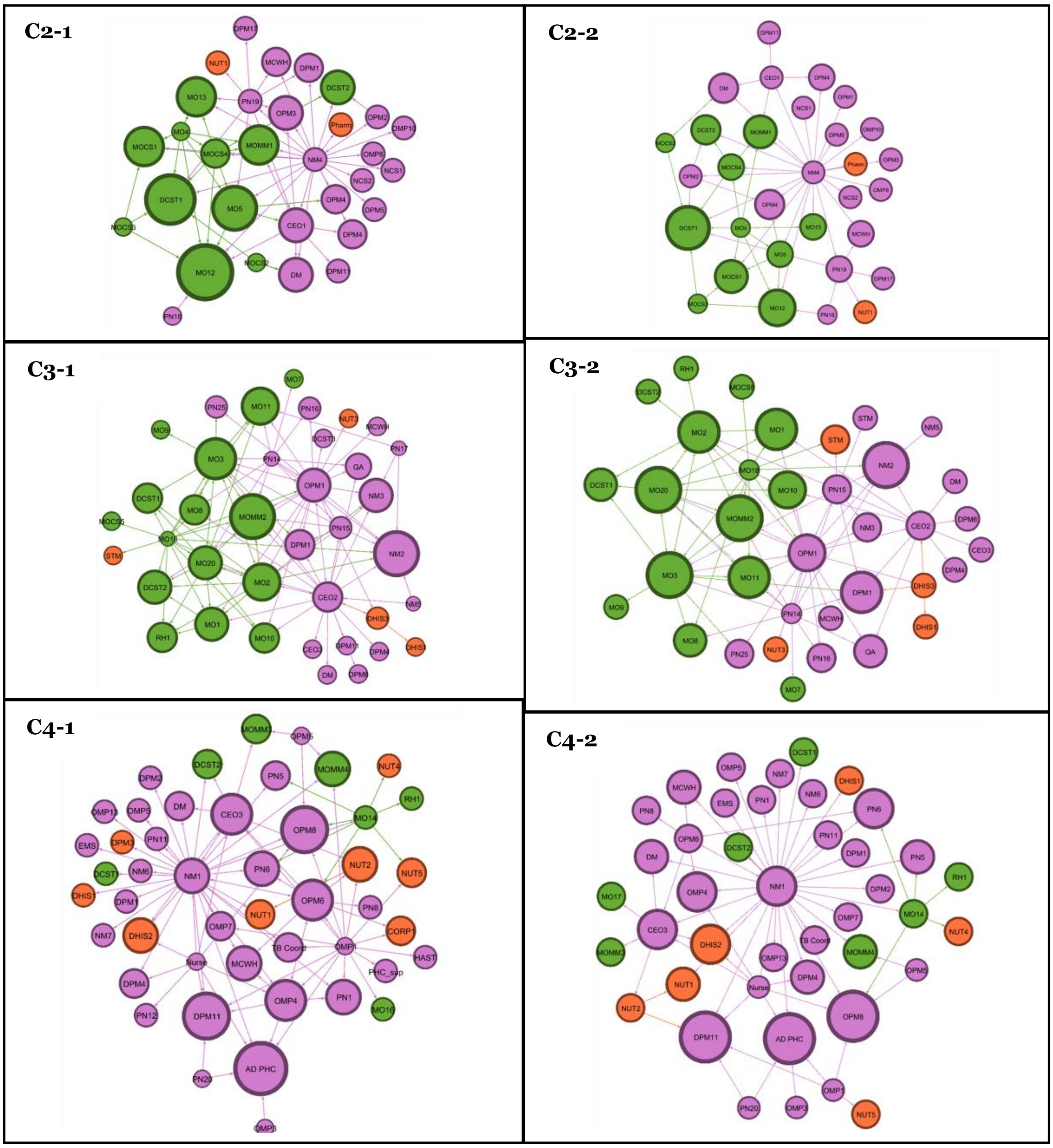

Legend – By Profession

Doctor

Nurses

Other

Figure S3: Sub-district clusters - Problem-solving (C2-1, C3-1, C4-1) and Sharing new idea (C2-2, C3-2, C4-2) networks
